# Baicalin Inhibits Biofilm Formation and the Quorum-Sensing System by Regulating the MsrA Drug Efflux Pump in *Staphylococcus saprophyticus*

**DOI:** 10.3389/fmicb.2019.02800

**Published:** 2019-12-10

**Authors:** Jinli Wang, Haihong Jiao, Jinwu Meng, Mingyu Qiao, Hongxu Du, Miao He, Ke Ming, Jiaguo Liu, Deyun Wang, Yi Wu

**Affiliations:** ^1^MOE Joint International Research Laboratory of Animal Health and Food Safety, College of Veterinary Medicine, Nanjing Agricultural University, Nanjing, China; ^2^Institute of Traditional Chinese Veterinary Medicine, College of Veterinary Medicine, Nanjing Agricultural University, Nanjing, China; ^3^Key Laboratory of Tarim Animal Husbandry Science and Technology of Xinjiang Production & Construction Corps, College of Animal Science, Tarim University, Alar, China

**Keywords:** baicalin, efflux pump, biofilm, quorum sensing (QS), *Staphylococcus saprophyticus*

## Abstract

*Staphylococcus saprophyticus* (*S. saprophyticus*) is one of the main pathogens that cause serious infection due to its acquisition of antibiotic resistance. The efflux pump decreases antibiotic abundance, and biofilm compromises the penetration of antibiotics. It has been reported that baicalin is a potential agent to inhibit efflux pumps, biofilm formation, and quorum-sensing systems. The purpose of this study was to investigate whether baicalin can inhibit *S. saprophyticus* biofilm formation and the quorum-sensing system by inhibiting the MsrA efflux pump. First, the mechanism of baicalin inhibiting efflux was investigated by the ethidium bromide (EtBr) efflux assay, measurement of ATP content, and pyruvate kinase (PK) activities. These results revealed that baicalin significantly reduced the efflux of EtBr, the ATP content, and the activity of PK. Moreover, its role in biofilm formation and the *agr* system was studied by crystal violet staining, confocal laser scanning microscopy, scanning electron microscopy, and real-time polymerase chain reaction. These results showed that baicalin decreased biofilm formation, inhibited bacterial aggregation, and downregulated mRNA transcription levels of the quorum-sensing system regulators *agrA*, *agrC*, RNAIII, and *sarA*. Correlation analysis indicated that there was a strong positive correlation between the efflux pump and biofilm formation and the *agr* system. We demonstrate for the first time that baicalin inhibits biofilm formation and the *agr* quorum-sensing system by inhibiting the efflux pump in *S. saprophyticus*. Therefore, baicalin is a potential therapeutic agent for *S. saprophyticus* biofilm-associated infections.

## Introduction

*Staphylococcus saprophyticus*, a member of the opportunistic coagulase-negative *Staphylococci* (CoNS), is a common pathogen of acute uncomplicated urinary tract infection, francolin ophthalmia, and bovine mastitis (Mahato et al., 2017; Mlaga et al., 2017; Wang J. et al., 2019). It has also been reported that *S. saprophyticus* is a common gastrointestinal flora in pigs and cows and may thus be transferred to humans by eating these respective foods. The use of antibiotics to treat and prevent bacterial infections has made an unprecedented impact on improving human health. However, multidrug-resistant bacteria have seriously threatened people’s health in recent decades. Wang et al. discovered that over 90% of *S. saprophyticus* isolated from ready-to-eat food displayed multiantibiotic resistance (Wang Y. et al., 2019). Bacteria are resistant to antibiotics due to target mutations, multidrug efflux pumps, drug-inactivating enzymes, biofilm formation, etc. Recently, *S. saprophyticus* has been shown to form biofilms in umbilical catheters (Martins et al., 2019).

Bacterial biofilms are adherent complex communities of bacteria encased within extracellular polymeric substances (EPSs), and they are considered intrinsically resistant to antibiotic treatment and host defenses (Thurlow et al., 2011; Paharik and Horswill, 2016). It has been estimated that nearly 60% of nosocomial infections in the human body are the result of biofilm formation on medical devices such as indwelling catheters and prostheses (Hou et al., 2012). The MIC of antibiotics toward bacteria biofilm is 1000-fold higher than that of planktonic counterparts (Costerton et al., 1987). Bacterial biofilms may represent an important barrier to the therapy of bacterial infections due to their lower sensitivity to antibiotic treatment. Therefore, the development of biofilm inhibitors based on a completely novel concept is urgently needed.

Recently, several studies have provided evidence to show that genetic inactivation and chemical inhibition of efflux pumps resulted in transcriptional inhibition of biofilm matrix components and a short biofilm formation (Baugh et al., 2014; Van Acker and Coenye, 2016; Sabatini et al., 2017). Biofilm formation is regulated by the quorum-sensing (QS) system. QS is a potential target for the therapy of bacterial biofilm infections. *Staphylococcus* uses a canonical Gram-positive two-component QS system encoded by the accessory gene regulator (*agr*) locus. Autoinducing peptide (AIP) is the signaling molecule of the *agr* system. When the cell density increases, the secretion of AIP is upregulated. In most Gram-positive QS bacteria, AIP is processed and exported by ABC transporters (Bassler, 1999). Our previous study indicated that azithromycin-resistant *S. saprophyticus* (ARSS) was resistant to macrolide antibiotics (Wang J. et al., 2019). The *ermA*, *ermB*, *ermC*, *mphC*, and *msrA* genes are the main resistance genes of macrolide antibiotics in *S. saprophyticus*, and the *msrA* gene is most prevalent in hospitals (Le Bouter et al., 2011). The MsrA efflux pump encoded by the *msrA* gene belongs to the ATP-binding cassette (ABC) transporters. We hypothesized that MsrA efflux pump inhibitors influence the *agr* system and biofilm formation.

**FIGURE 1:**
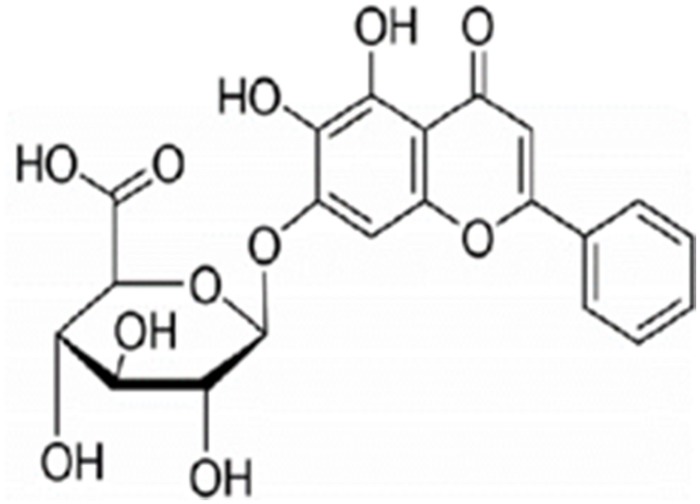
Chemical structure of baicalin (Moore et al., 2016).

Several natural plant products act as efflux pump inhibitors (Stavri et al., 2007). Baicalin (Figure 1; Moore et al., 2016), a type of flavonoid derived from the roots of *Scutellaria baicalensis* Georgi, exerts many biological activities and pharmacological effects, including remarkable antibiofilm, antibacterial (Sass et al., 2019), antiviral (Chen et al., 2018), and immune-enhancing ability (Ge et al., 2012). Our previous study has shown that baicalin possessed synergistic anti-ARSS with azithromycin (Azm; Wang J. et al., 2019). Chen et al. elucidated that a sub-inhibitory concentration of baicalein can prevent biofilm formation by inhibiting the *agr* system of *S. aureus* (Chen et al., 2016). Thus, we hypothesized that baicalin has the potential to be an effective therapeutic strategy against *S. saprophyticus* biofilm formation and the *agr* system by inhibiting efflux pumps.

Considering these aspects, this study evaluated whether baicalin could effectively inhibit the efflux pump, biofilm formation, and QS system in ARSS. We also tried to explain the relationship between the efflux pump and biofilm formation and the QS system. To our knowledge, there are no papers regarding the relationship between efflux and biofilm formation and the QS system in *S. saprophyticus.*

## Materials and Methods

### Bacterial Strains and Culture Conditions

The azithromycin-resistant *S. saprophyticus* (ARSS) strain used in this experiment was isolated in 2016 from francolins suffering from ophthalmia in a francolin farm in Jiangsu Province, China. ARSS was coagulase negative and resistant to azithromycin with an MIC value of 1000 mg/L (Wang J. et al., 2019). *S. saprophyticus* ATCC 15305 was purchased from the China Center of Industrial Culture Collection (CICC). Strains were routinely cultured on brain heart infusion broth (BHI; Haibo, Qingdao, China) or nutrient broth (NB, Haibo, Qingdao, China) and incubated at 37°C.

### Determination of Growth Kinetics

For the growth kinetics assay, overnight ARSS cultures were prepared and cocultured with sub-inhibitory concentrations of baicalin or verapamil (an efflux pump inhibitor as a positive control compound). Normal saline containing up to 2% NaHCO_3_ was used to prepare a 4000 mg/L baicalin solution. We have previously demonstrated that the MIC value of ARSS to baicalin was 500 mg/L (Wang J. et al., 2019). The sub-inhibitory concentrations of baicalin were used in this experiment, and the final concentrations of baicalin were 250, 125, 62.5, and 31.25 mg/L. The final concentration of verapamil was 250 mg/L. At the same time, the *S. saprophyticus* control group (SS group, not containing drug) was set. The cell densities based on the optical density at 600 nm in BHI were measured every 2 h from 0 h to 24 h.

### Efflux of Ethidium Bromide

The efflux of ethidium bromide (EtBr) by ARSS was implemented as previously described with some modifications (Smith and Blair, 2014): bacterial strains were grown to an OD_600 nm_ of 0.6. The cells were pelleted by centrifuging at 4000 rpm for 10 min at room temperature, and the precipitation was washed twice with the same volume of phosphate buffer saline (PBS). Then, the OD_600 nm_ of the bacterial suspension was adjusted to 0.3 with PBS containing 1 mM MgCl_2_. EtBr was added at a final concentration of 7.81 mg/L (1/2 MIC). Cultures were incubated at 37°C with shaking for 60 min. After centrifugation for 10 min at 4000 rpm, the supernatant was discarded, and the pellet was resuspended in PBS with 1 mM MgCl_2_ and 5% glucose to energize the cells. Aliquots of 0.05 ml were put into each well of black, clear-bottom 96-well microplates that contained 0.05 ml baicalin or verapamil at sub-inhibitory concentrations prepared at 2-fold serial dilutions in PBS with 1 mM MgCl_2_ and 5% glucose. Verapamil at 250 mg/L was used as a known efflux pump inhibitor, while the *S. saprophyticus* control (SS) group contained an equal volume of PBS with 1 mM MgCl_2_ and 5% glucose. The fluorescence was measured over 60 min at excitation and emission wavelengths of 530 nm and 590 nm, respectively, in a Tecan Infinite 200 Pro (Switzerland).

### Measurement of the ATP Content and Pyruvate Kinase Activity

Overnight cultures were used to inoculate 4 ml of NB containing baicalin at concentrations of 250, 125, 62.5, and 31.25 mg/L and incubated at 37°C with shaking (180 rpm). The samples from each group were removed at 24 h. Then, the ATP content and pyruvate kinase (PK) activity were determined using an ATP assay kit (Beyotime, China) and a Pyruvate Kinase Assay Kit (Solarbio, Beijing, China) according to the manufacturer’s instructions.

### Measuring Gene Transcription Levels via RT-PCR

Overnight cultures were used to inoculate 4 ml of NB containing baicalin at concentrations of 250, 125, 62.5, and 31.25 mg/L and incubated at 37°C with shaking (180 rpm). The samples used to detect the transcription level of the *msrA* gene from each group were removed at 24 h in the efflux mechanism experiment. Overnight cultures of ARSS were diluted into fresh BHI. Then, 0.5 ml of diluted cultures was applied to each well of sterile 96-well flat-bottom tissue culture plates that contained an equal volume of sub-inhibitory concentrations of baicalin (final concentrations of 250, 125, 62.5, and 31.25 mg/L) or verapamil (final concentration of 250 mg/L). The *S. saprophyticus* control (SS) group contained an equal volume of fresh BHI. The plates were incubated at 37°C without shaking. The samples from each group were removed at 24 h and 48 h post-inoculation for RNA extraction. These samples were used to detect the transcription levels of *msrA*, *agrA*, *agrC*, RNAIII, and *sarA* in biofilms. Then, total RNA was extracted by using a Bacteria RNA kit (Vazyme, Nanjing, China) as recommended in the manufacturer’s instructions. The value of A260/A280 was confirmed to be between 1.8 and 2.1. The reverse transcription assay took place in a PCR instrument (2720 Thermal Cycler PCR instrument, Applied Biosystems, America) by using a HiScript II 1st Strand cDNA Synthesis Kit (Vazyme, Nanjing, China). Reverse transcription was carried out at 50°C for 15 min and 85°C for 5 s. Real-time PCR was reacted in a PCR instrument (StepOnePlus^TM^ Real Time PCR instrument, Applied Biosystems, United States) using ChamQTM SYBR^®^ qPCR Master Mix according to the manufacturer’s instructions. The cycling parameters were as follows: holding stage of 95°C for 3 min; 40 cycles at the cycling stage of 95°C for 10 s and 60°C for 60 s; one melt curve stage of 95°C for 15 s, then 60°C for 60 s, and 95°C for 15 s. The 16S rRNA of ARSS was chosen as the housekeeping control gene. The sequences of primers used in this experiment are listed in Table 1.

**TABLE 1:** Oligonucleotide primers used in this study.

**Target gene**	**Primer**	**Sequence (5′–3′)**	**Source**
16S rRNA	16S rRNA-F	TGAAGAGTTTGATCATGGCTCAG	Hwang et al., 2011
	16S rRNA-R	ACCGCGGCTGCTGGCAC	
msrA	msrA-F	GCTCTACTGAATGATTCTGATG	This study
	msrA-R	TGGCATACTATCGTCAACTT	
agrA	agrA-F	CCACTGCTGATCCTTATGA	This study
	agrA-R	GCGGCTACCTTATAGACAA	
agrC	agrC-F	GTCATTACACCACTGCTATTC	This study
	agrC-R	GTCCATCCATATCTTCTTCTCT	
sarA	sarA-F	ATTAGCGATGGTTACTTACG	This study
	sarA-R	CTGCTTTAACAACTTGAGGT	
RNAIII	RNAIII-F	ACGACCTTCACTTGTATCC	This study
	RNAIII-R	GCTACGGCATCTTCTTCTA	

### Semiquantitative Determination of Biofilm Formation

Semiquantitative biofilm assays were conducted as described previously with some modifications (Liu et al., 2017). Briefly, overnight cultures of ARSS or ATCC 15305 strains were diluted into fresh BHI. Then, 0.1 ml of diluted cultures was applied to each well of sterile 96-well plates that contained an equal volume of sub-inhibitory concentrations of baicalin (final concentrations of 250, 125, 62.5, and 31.25 mg/L) or verapamil (final concentration of 250 mg/L). The *S. saprophyticus* control (SS) group and the ATCC group contained equal volumes of fresh BHI. The negative control group contained only an equal volume of fresh BHI. Then, the plates were incubated at 37°C for 24 and 48 h without shaking. Culture supernatants were gently removed, and wells were washed with PBS twice to remove the floating cells, followed by fixation with 2.5% glutaraldehyde for 1.5 h and finally air-dried. The adherent bacteria in the wells were stained for 20 min with 1% (wt/vol) crystal violet and then rinsed thoroughly with PBS until the negative control wells (without biofilms) appeared colorless. To quantify biofilm formation, 0.2 ml of 33% glacial acetic acid was added to the wells of plates that were stained with crystal violet. Biofilm formation was measured with a Thermo^TM^ Multiskan^TM^ FC enzyme-labeled instrument at 570 nm. The results are presented as the values in the experimental groups minus the values in the negative control group.

### CLSM Protocol Studies for Biofilm Inhibition

Biofilms were stained with the fluorescent LIVE/DEAD BacLight^TM^ bacterial viability kit L7012 (Molecular Probes, Invitrogen) according to the manufacturer’s instructions. Bacteria with damaged or intact cell membranes stain fluorescent green when SYTO 9 was used alone. Briefly, fluorescent green (SYTO 9) was used according to the product information manual supplied by the manufacturer. Afterward, 1 ml of overnight cultures that were diluted by BHI was used to grow biofilms on cover slides in 6-well microtiter plates containing 1 ml of baicalin at concentrations of 62.5, 125, 250, and 500 mg/L, verapamil (VP group) at a concentration of 500 mg/L or an equal volume of BHI (SS group) for 24 or 48 h at 37°C without shaking. Then, the supernatant was removed, and the adherent organisms were stained with dye at room temperature in the dark for 15 min. Finally, these carriers were rinsed three times with 0.85% NaCl and detected under CLSM (Nikon A1).

### Scanning Electron Microscope Studies for Biofilm Morphology

Biofilm morphology was observed using an S3400N scanning electron microscope (SEM; Hitachi, Japan). In this experiment, 0.5 ml of overnight cultures that were diluted with fresh BHI were used to grow biofilms on round cover slides in 24-well microtiter plates containing 0.5 ml of baicalin at a concentration of 500 mg/L or an equal volume of BHI (SS group) for 48 h at 37°C without shaking. Each biofilm slice was washed with PBS, fixed in 2.5% glutaraldehyde overnight at 4°C, and then rinsed thoroughly three times with fresh PBS (pH 7.4). The slices were passed through an ethanol gradient (e.g., 50, 70, 80, and 90%) for 15 min, passed through 100% ethanol (three times for 10 min) for dehydration, dried, and then coated with gold (Fujimura et al., 2008).

### Baicalin and Azithromycin Combined Efficacy *in vitro* on Mature Biofilm

In order to compare the combined effects of baicalin and Azm against ARSS biofilms, 48 h biofilms were prepared in 24-well plates. Briefly, overnight cultures of ARSS were diluted into fresh BHI. Then, 1 ml of diluted cultures was applied to each well of sterile 96-well flat-bottom tissue culture plates. The plates were incubated at 37°C for 48 h without shaking. Then, the culture supernatants were gently removed. The plates were gently washed twice with PBS. Baicalin and verapamil in the presence or absence of Azm were added to each well in BHI. The total volume per well was 1 ml. The final concentrations of verapamil and Azm were 250 and 7.8 mg/L, respectively. The final concentrations of baicalin were 250, 125, 62.5, and 31.25 mg/L. Finally, the plates with biofilms were cultured for 24 h at 37°C. After the treatment was completed, planktonic bacteria were discarded by washing with PBS, and clumps were disrupted by sonicating. The bacterial CFU counts in biofilm were performed by plating serial dilutions on NB agar. This experiment was conducted three times in parallel.

### Correlation Analysis

Correlations among the relative expression of the efflux gene and the ability of biofilm formation and the relative expression of *agr* system-associated genes were determined using Pearson’s correlation coefficient.

### Statistical Analysis

Relative gene expression data were analyzed by the 2^–ΔΔ*CT*^ method. Duncan’s Multiple Range Test was used to determine the differences among groups by the SPSS Software Package version 20.0 (IBM, Armonk, NY, United States). The results were expressed as the mean ± standard deviation (SD). Differences were considered statistically significant at *p* < 0.05.

## Results

### Sub-Inhibitory Concentrations of Baicalin and Verapamil Did Not Influence Bacterial Growth *in vitro*

**FIGURE 2:**
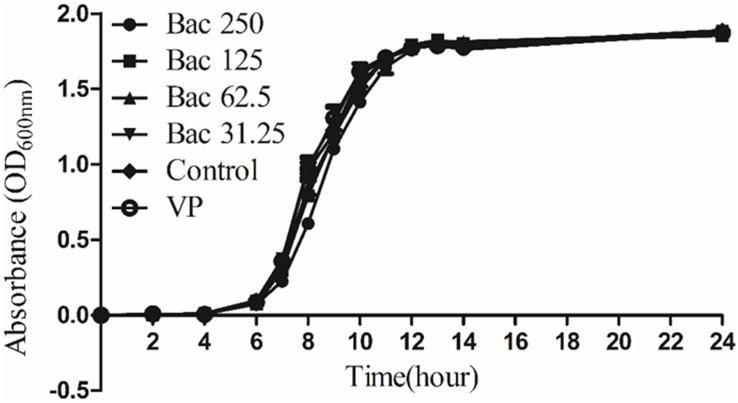
Growth kinetics of *Staphylococcus saprophyticus* strains in BHI under sub-inhibitory concentrations of baicalin or verapamil. OD_600 nm_ values are the means ± SD from three independent experiments. Sub-inhibitory concentrations of baicalin are 250, 125, 62.5, and 31.25 mg/L, and verapamil had no effect on bacterial growth.

Our previous investigation indicated that the MICs of baicalin and azithromycin against ARSS were 500 and 1000 mg/L, respectively (Wang J. et al., 2019). The results of the growth kinetics assay showed that the growth kinetics of the ARSS under sub-inhibitory concentrations of baicalin and verapamil did not exhibit any difference from the control group when the bacteria were cultured in BHI, indicating that in a nutrient-rich environment, the sub-inhibitory concentrations of baicalin and verapamil may not affect *S. saprophyticus* growth (Figure 2).

### Baicalin Inhibited EtBr Efflux

**FIGURE 3:**
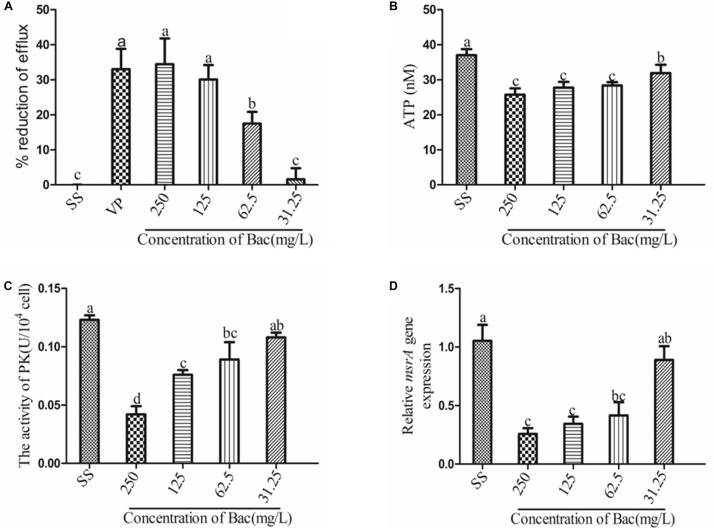
Baicalin-mediated ARSS efflux. **(A)** Baicalin and positive control verapamil inhibited the efflux of EtBr in ARSS. **(B)** Baicalin influenced the ATP content. **(C)** Baicalin influenced the activity of PK. **(D)** Baicalin influenced the transcription level of the *msrA* gene. Bars in the same index without the same letters differ significantly (*p* < 0.05).

The inhibitory effect of increasing concentrations of baicalin and the known efflux pump inhibitor verapamil (VP) on EtBr efflux is shown in Figure 3. A concentration-dependent effect was observed for baicalin. In the presence of verapamil or 250, 125, or 62.5 mg/L baicalin, the rate of efflux reduction was significantly higher than that of the SS group, as presented in Figure 3A (*p* < 0.05). Importantly, the rates of efflux reduction were not significantly different from that of the VP group when 250 or 125 mg/L baicalin was added. MsrA is an ATP-dependent efflux pump, suggesting that ATP is essential for MsrA efflux function (Hu et al., 2015). PK is associated with ATP production. Thus, the ATP content and PK activity were detected. Baicalin markedly decreased the content of ATP and the activity of PK (Figures 3B,C) (*p* < 0.05). The MsrA efflux pump was encoded by the *msrA* gene. Baicalin significantly reduced the relative expression of the *msrA* gene at 250, 125, and 62.5 mg/L, as presented in Figure 3D (*p* < 0.05). However, baicalin at 31.25 mg/L did not exert beneficial effects.

### Biofilm Formation Was Impeded by Baicalin and Verapamil

**FIGURE 4:**
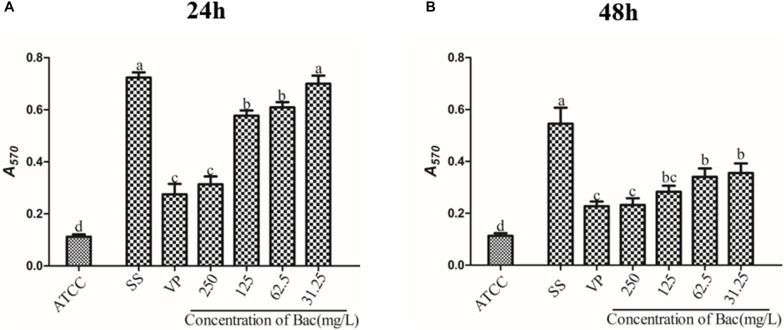
The influence on the biofilm formation ability of baicalin and verapamil. The crystal violet assay assessed the biomass of ATCC 15305 and ARSS after exposure to 250, 125, 62.5, and 31.25 mg/L baicalin and 250 mg/L verapamil for 24 h **(A)** and 48 h **(B)**. Bars in the same index without the same letters differ significantly (*p* < 0.05).

**FIGURE 5:**
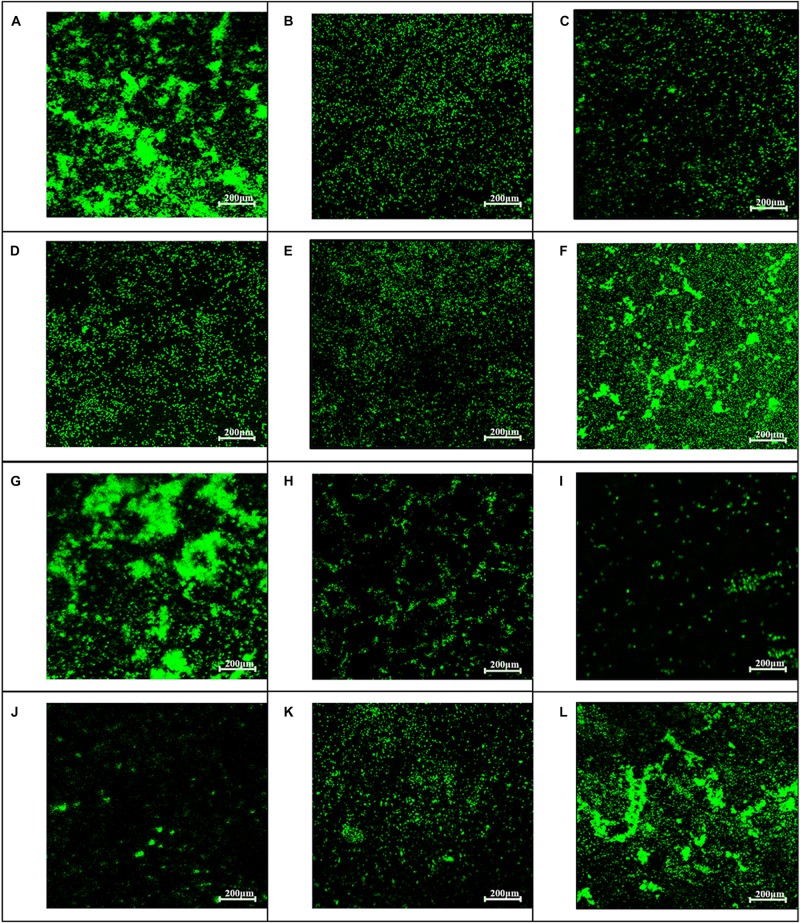
Influence of baicalin on biofilm formation assessed by fluorescence microscopy (200×). Static biofilms after exposure to baicalin or verapamil for 24 h **(A–F)** and 48 h **(G–L)** were stained with SYTO 9. ARSS within biofilms on glass carriers display green fluorescence. Control group **(A,G)**; verapamil group **(B,H)**; 250 mg/L baicalin group **(C,I)**; 125 mg/L baicalin group **(D,J)**; 62.5 mg/L baicalin group **(E,K)**; 31.25 mg/L baicalin group **(F,L)**.

**FIGURE 6:**
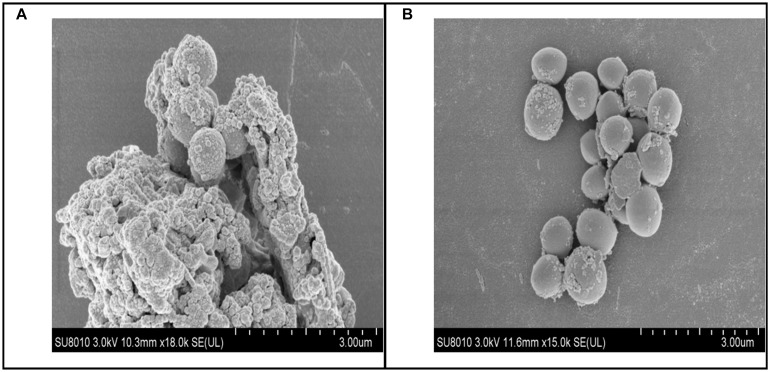
Biofilm scanning by SEM. **(A)** Bacteria control group, lots of bacteria aggregated together and enrolled by EPS. **(B)** 250 mg/L baicalin group, a small amount of bacteria aggregated.

To examine whether baicalin or verapamil inhibited ARSS biofilm formation, the ability of biofilm formation was investigated. These results reflected that the *A*_570_ values of ARSS biofilm in the SS group were significantly higher than those of the ATCC group for 24 and 48 h. We found that baicalin reduced biomass in a dose-dependent manner in spite of whether the cultures were grown for 24 or 48 h, while 31.25 mg/L baicalin had almost no effect at 24 h in ARSS (Figures 4A,B). Additionally, verapamil significantly decreased biofilm formation compared with that of the SS group (not containing drug). Interestingly, the *A*_570_ values were not different when verapamil or 250 mg/L baicalin was added for 24 or 48 h. Moreover, fluorescence microscopy revealed similar results (Figure 5). From Figures 5, 6, we find that many bacteria accumulated together and were enrolled in large amounts of extracellular matrix in the SS group. However, when baicalin was added, only a few bacteria adhered to the glass side and did not form mature biofilms.

### Combined Anti-ARSS Biofilm Efficacy of Baicalin Plus Azm

**FIGURE 7:**
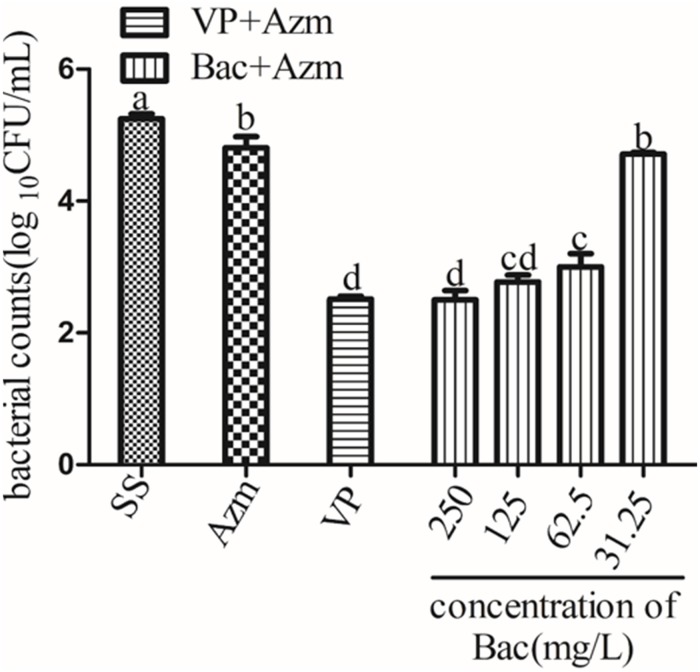
Viable bacterial counts of 48 h biofilms after exposure to agents for 24 h. Biofilms were formed on slices for 48 h by growing *ARSS* in BHI and then treated with Azm alone or in combination with 250, 125, 62.5, and 31.25 mg/L baicalin and 250 mg/L verapamil for 24 h. Bars in the same index without the same letters differ significantly (*p* < 0.05).

To determine whether baicalin was effective at disrupting ARSS biofilms combined with Azm, 48-h biofilms were treated with baicalin and verapamil in the presence or absence of Azm. The results showed that Azm alone did not eradicate ARSS in the formed biofilms. However, combination treatment with verapamil + Azm or baicalin + Azm markedly reduced the counts of bacteria on plates in a concentration-dependent manner (*p* < 0.05), while 31.25 mg/L baicalin had almost no effect. Furthermore, there was no significant difference between the 250 and 125 mg/L baicalin + Azm groups and the verapamil + Azm group (Figure 7).

### Baicalin Inhibited the Relative Expression Level of *the msrA* Efflux Gene in Biofilm

**FIGURE 8:**
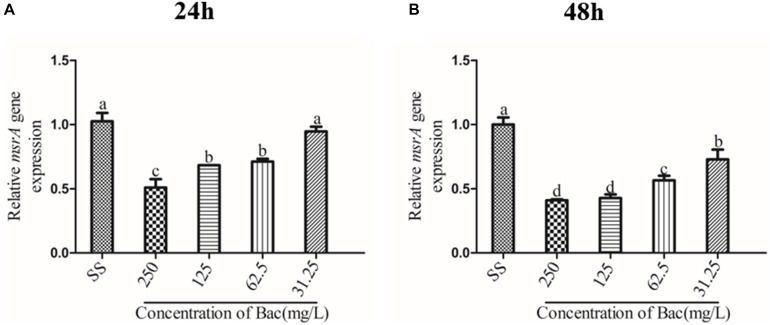
Influence of *msrA* gene relative expression in biofilm bacteria of baicalin (using 16S rRNA as the internal parametric gene). The relative expression of the *msrA* gene was determined in samples prepared from 24 h **(A)** and 48 h **(B)** ARSS biofilms in BHI using quantitative RT-PCR. Data are derived from three biological repeats. Bars in the same index without the same letters differ significantly (*p* < 0.05).

We measured the expression of the *msrA* gene in ARSS biofilm bacteria at 24 and 48 h by RT-PCR to detect the relationship between efflux and biofilm formation. As shown in Figure 8, the relative expression of the *msrA* gene was significantly decreased in a dose-dependent manner when baicalin was added compared with relative expression in the SS group (*p* < 0.05). However, the transcript level of the *msrA* gene was not significantly affected by 31.25 mg/L baicalin at 24 h.

### In the ARSS Background, Baicalin and Verapamil Lowered the Activity of the *agr* System

**FIGURE 9:**
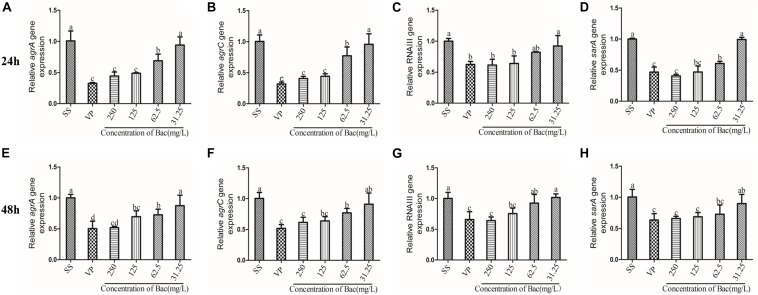
Relative expression of the *agr* system-associated genes (using 16S rRNA as the internal parametric gene). The expression levels of *agr*A **(A,E)**, *agrC* **(B,F)**, *RNA*III **(E,G)**, and *sarA* **(D,H)** were determined in samples prepared from 24 h **(A–D)** and 48 h **(E–H)** ARSS biofilms in BHI using quantitative RT-PCR. Data are derived from three biological repeats. Bars in the same index without the same letters differ significantly (*p* < 0.05).

In *Staphylococcus*, the two-component QS system *agr* is an important contributor to the establishment of biofilm and infection by this bacterium (Boles and Horswill, 2008). In order to detect the transcription levels of specific RNA in different samples, the levels of *agr* system-associated gene transcripts were measured using RT-PCR. After the bacterial biofilms were treated with either 250, 125, or 62.5 mg/L baicalin or 250 mg/L verapamil for 24 and 48 h, the transcription levels of *agrA*, *agrC*, *sarA*, and RNAIII genes significantly decreased in a dose-independent manner (*p* < 0.05). Treating the biofilms with 31.25 mg/L baicalin (24 or 48 h) did not change *agr* system-associated gene expression levels. More importantly, no significant difference in *agr* system-associated gene expression levels resulted from treatment with 250 mg/L baicalin and verapamil (Figure 9).

### Pearson’s Correlation Coefficients Between the Relative Expression of the Efflux Gene and Biofilm Formation and the Transcript Levels of *agr* System-Associated Genes

The correlation coefficients among the measured indices of *msrA* efflux gene relative expression and biofilm formation and transcript levels of *agr*-associated genes are presented in Table 2. As shown in Table 2, the relative expression of the *msrA* efflux gene was positively correlated with the *A*_570_ values of biofilm (*p* < 0.01) and the relative expression of *agr* system-associated genes (*p* < 0.01). In particular, the relative expression of *agrA* and *agrC* was positively correlated with the relative expression of *sarA* and RNAIII.

**TABLE 2:** Pearson’s correlation coefficients between the relative expression of *msrA* and biofilm formation ability and the expression of *agr* system-associated genes.

	**BF formation**	***msrA***	***agrA***	***agrC***	***sarA***	***RNAIII***
BF formation	1	0.850^∗∗^	0.584	0.560	0.549	0.480
*msrA*		1	0.814^∗∗^	0.769^∗∗^	0.764^∗^	0.723^∗^
*agrA*			1	0.927^∗∗^	0.937^∗∗^	0.943^∗∗^
*agrC*				1	0.924^∗∗^	0.943^∗∗^
*sarA*					1	0.803^∗∗^
*RNAIII*						1

**^∗∗^p* < 0.01, *^∗^p* < 0.05. 0.8–1.0, extreme correlation; 0.6–0.8, strong correlation; 0.4–0.6, moderate correlation; 0.2–0.4, weak correlation; 0.0–0.2, very weak correlation or no correlation.*

## Discussion

Efflux pumps, which are membrane-bound proteinaceous transporters, are in charge of transporting xenobiotics or chemotherapeutic agents that are otherwise harmful for bacterial survival. Many researchers have reported that multidrug-resistant (MDR) efflux pumps play a prominent role in the biology of bacteria and have roles in drug resistance, cell division, pathogenicity, and, as recently described, the formation of biofilms (Kvist et al., 2008; Buckley et al., 2006). MsrA is an MDR efflux pump of the ABC family. It was established to expel various macrolide antibiotics in *Staphylococcus*. The MsrA efflux pump has been well established as a model system for studying efflux inhibition in *Staphylococcus* (Chan et al., 2013).

In the present study, baicalin was assessed for its MsrA efflux pump inhibitory activity in ARSS, which expresses the *msrA* efflux gene and is resistant to Azm. Baicalin could inhibit 33% of the EtBr extrusion from ARSS. Importantly, there was no significant difference in the rate of efflux reduction resulting from treatment with either the positive control compound verapamil or 250 mg/L baicalin (Figure 3A). These results suggested that baicalin could inhibit the MsrA efflux pump and has the potential to be an efflux pump inhibitor. MsrA is an ATP-dependent efflux pump, indicating that the inhibitory activities of baicalin against the ARSS efflux pump were more correlated with the ATP-dependent process in bacteria (Hu et al., 2015). In this study, our results indicated that baicalin significantly decreased the content of ATP compared with the SS group. PK, a final-stage enzyme in glycolysis, converts phosphoenolpyruvate (PEP) to pyruvate and ATP (Zoraghi et al., 2010). PK is critical for bacterial survival. Chan et al. proved that diosmetin could statistically reverse the resistance of MRSA to erythromycin possibly by inhibiting the MsrA efflux pump *in vitro* and MRSA-specific PK selectively, and they also confirmed that baicalein could inhibit the activity of PK (Chan et al., 2013; Chan et al., 2011). Because the chemical structure of diosmetin is similar to baicalin, it is interesting to detect whether baicalin has potential inhibitory actions on ARSS PK. In this study, our results revealed that the enzymatic activity of ARSS PK was inhibited by baicalin. It is possible that interfering with ATP production by baicalin may stop the function of the MsrA efflux pump and contribute to the synergistic action of baicalin and Azm against ARSS. We also elucidated that baicalin could dose-dependently inhibit the transcript level of the *msrA* gene. Taken together, these results indicated that baicalin interfered with ATP generation by inhibiting PK enzymatic activity and decreasing the relative expression of the *msrA* gene, ultimately inhibiting the MsrA efflux pump.

It has been reported that increasing resistance is associated with active efflux in bacterial biofilms (Van Acker and Coenye, 2016; Danquah et al., 2018). Baugh et al. demonstrated that numerous functional efflux pumps give rise to biofilm matrix expression in *Salmonella Typhimurium* and that the addition of a variety of efflux inhibitors inhibited biofilm formation (Baugh et al., 2012). Therefore, we hypothesized that the efflux inhibitor baicalin could be used as a biofilm formation inhibitor. In the present study, sub-inhibitory concentrations of baicalin or positive control verapamil did not affect *S. saprophyticus* growth (Figure 2) but reduced the values of *A*_570_ measured by staining with crystal violet. ARSS was unable to form mature biofilms under baicalin or verapamil incubation conditions even after extended incubation times up to 48 h. These results indicated that baicalin and verapamil inhibited biofilm formation. Pearson’s correlation coefficient analysis indicated that the relative expression of the efflux gene was positively correlated with biofilm formation (*p* < 0.01). Therefore, baicalin can inhibit biofilm formation by inhibiting the MsrA efflux pump in ARSS. Evidently, biofilms render the cells less accessible to the defense system of the organism and reduce antibiotic concentrations inside the target pathogen. The treatment of biofilm-related infections is a critical clinical problem in the current era. Because of frequent reports on resistance to antimicrobials, the synergistic antibacterial method may be a better treatment strategy (Drugeon et al., 1999). The combined action of molecules was reported to eradicate the biofilms (Baugh et al., 2014). Overall, this evidence encouraged the investigation of the roles of baicalin in the prevention and eradication of biofilms. In this study, baicalin and Azm increased antibiotic permeability by disrupting the already-formed biofilms. Verapamil was used as a positive control compound to evaluate the combined effects of sub-inhibitory concentrations of baicalin with Azm on biofilms that were established for 48 h. *S. saprophyticus* could not be eradicated by Azm alone, and both baicalin + Azm and VP + Azm could significantly decrease the counts of bacteria compared with the counts for the SS group. Presumably, baicalin may be a potential agent for treating biofilm infections by either inhibiting biofilm formation or eradicating biofilms in combination with Azm.

In addition, multidrug efflux pumps often secrete metabolites involved in QS (Polkade et al., 2016). QS is a process of bacterial cell–cell communication that allows bacteria to sense cell density and change bacterial gene expression patterns to alter bacteria group behaviors at high cell numbers (Rutherford and Bassler, 2012; Camilli and Bassler, 2006). This cross-talk between bacteria is believed to be essential for the establishment of bacterial biofilms and bacterial biofilm infection (Danquah et al., 2018). QS regulates the expression of genes encoding virulence factors involved in a range of toxins, adhesion molecules, and compounds that influence immune function. Chen et al. (2016) prophase studies demonstrated that baicalein interfered with the QS system and affected bacterial virulence. Therefore, the potential target for the treatment of bacterial biofilm infection is the QS system. *Staphylococcus* biofilm is regulated by a Gram-positive two-component QS system encoded by the *agr* locus. An increase in cell density accounts for a prompt upsurge in the production, secretion, and detection of AIP. When AIP accumulates, it binds with *agrC*, which is a membrane-bound histidine kinase. Then, *agrC* autophosphorylates at a conserved histidine and transfers the phosphate group to an aspartate on the response regulator *agrA* (Lina et al., 1998). *AgrA* activates the divergently encoded P3 promoter, which controls the expression of RNAIII (Novick et al., 1993). Most of the effects of QS regulating virulence in *Staphylococcus* are achieved by direct and indirect regulation of RNAIII (Queck et al., 2008). Our results showed that 250, 125, and 62.5 mg/L baicalin and verapamil downregulated the transcript levels of *agrA*, *agrC*, RNAIII, and *sarA* in a dose-dependent manner compared with that of the SS group. In most Gram-positive QS bacteria, AIP was processed and exported by ABC transporters (Bassler, 1999). The MsrA efflux pump belongs to the ABC transporters. We found that there was a significant correlation between the relative expression of *agr* system-associated genes and the efflux gene (*p* < 0.01, Table 2). Therefore, we inferred that baicalin may inactivate the *agr* system by inhibiting the efflux of AIP. Recent findings (Ge et al., 2012; Drugeon et al., 1999) have demonstrated that RNAIII regulates biofilm formation and induces toxin production, such as plasma-coagulase, enterotoxin, and thermostable nuclease. Additionally, RNAIII is a transcriptional regulator of the *Staphylococcal* accessory regulator A (*sarA*) family (Lewis, 2007). Recent evidence has elucidated that SarA, as a central regulatory element, controls the production of staphylococcus virulence factors (Van Acker and Coenye, 2016). Therefore, we inferred that baicalin may regulate virulence by inhibiting the agr system.

## Conclusion

In conclusion, baicalin effectively inhibited the MsrA efflux pump, biofilm formation and the *agr* system in ARSS. In addition, there is a significant positive correlation between efflux and biofilm formation and the *agr* system. Therefore, we believe that *S. saprophyticus* biofilm-related infections could be treated by baicalin combined with Azm. To our knowledge, we first elucidated the positive relationship between efflux and biofilm formation in *S. saprophyticus*.

## Data Availability Statement

All datasets generated for this study are included in the article.

## Author Contributions

JW, HJ, and JL conceived and designed the experiments. JW, JM, MQ, HD, MH, and KM performed the experiments. JW and HJ analyzed the data. DW and YW contributed to the reagents, materials, and analysis tools. JW wrote the manuscript. All authors read and made additions to the manuscript during revision stages.

## Conflict of Interest

The authors declare that the research was conducted in the absence of any commercial or financial relationships that could be construed as a potential conflict of interest.
